# Reduced Hippocampal Volume and Neurochemical Response to Adult Stress Exposure in a Female Mouse Model of Urogenital Hypersensitivity

**DOI:** 10.3389/fpain.2022.809944

**Published:** 2022-01-27

**Authors:** Aaron D. Brake, Xiaofang Yang, Chu-Yu Lee, Phil Lee, Paul Keselman, Olivia C. Eller, In-Young Choi, Janna L. Harris, Julie A. Christianson

**Affiliations:** ^1^Department of Anatomy and Cell Biology, University of Kansas Medical Center, Kansas City, KS, United States; ^2^University of Kansas Medical Center, Hoglund Biomedical Imaging Center, Kansas City, KS, United States; ^3^Department of Molecular and Integrative Physiology, University of Kansas Medical Center, Kansas City, KS, United States; ^4^Department of Radiology, University of Kansas Medical Center, Kansas City, KS, United States; ^5^Department of Neurology, University of Kansas Medical Center, Kansas City, KS, United States; ^6^Department of Anesthesiology, Pain, and Perioperative Medicine, University of Kansas Medical Center, Kansas City, KS, United States

**Keywords:** magnetic resonance imaging, magnetic resonance spectroscopy, early life stress, pain, obesity

## Abstract

Early life stress exposure significantly increases the risk of developing chronic pain syndromes and comorbid mood and metabolic disorders later in life. Structural and functional changes within the hippocampus have been shown to contribute to many early life stress-related outcomes. We have previously reported that adult mice that underwent neonatal maternal separation (NMS) exhibit urogenital hypersensitivity, altered anxiety- and depression-like behaviors, increased adiposity, and decreased gene expression and neurogenesis in the hippocampus. Here, we are using magnetic resonance imaging and spectroscopy (MRI and MRS) to further investigate both NMS- and acute stress-induced changes in the hippocampus of female mice. Volumetric analysis of the whole brain revealed that the left hippocampus of NMS mice was 0.038 mm^3^ smaller compared to naïve mice. MRS was performed only on the right hippocampus and both total choline (tCho) and total N-acetylaspartate (tNAA) levels were significantly decreased due to NMS, particularly after WAS. Phosphoethanolamine (PE) levels were decreased in naïve mice after WAS, but not in NMS mice, and WAS increased ascorbate levels in both groups. The NMS mice showed a trend toward increased body weight and body fat percentage compared to naïve mice. A significant negative correlation was observed between body weight and phosphocreatine levels post-WAS in NMS mice, as well as a positive correlation between body weight and glutamine for NMS mice and a negative correlation for naïve mice. Together, these data suggest that NMS in mice reduces left hippocampal volume and may result in mitochondrial dysfunction and reduced neuronal integrity of the right hippocampus in adulthood. Hippocampal changes also appear to be related to whole body metabolic outcomes.

## Introduction

According to the National Survey of Children's Health, nearly 40% of children in the United States experienced at least one or more serious psychological traumas in 2018 (1). Exposure to early life stress (ELS) has a significant and long-lasting impact on health outcomes later in life, including chronic pain, mood, and metabolic disorders (2) and a higher rate of comorbidity among these outcomes (3–8). Experiencing multiple adverse childhood events (ACEs) can permanently alter higher level affective processing (6) and is associated with psychiatric disorders including depression and anxiety (9). A growing base of research looking into the effects of ELS on pain processing has shown a heightened sensitization to painful stimuli, an increased likelihood of developing chronic pain syndromes, and a decreased likelihood of symptom improvement over time (7, 10, 11).

Many of the long-term effects of ELS are believed to be mediated via impaired development of the hippocampus. Excess exposure to glucocorticoids during early development, either through pharmacological or physiological sources, significantly impairs the structure, function, and gene expression of the hippocampus (12). Considering that the hippocampus is a major negative regulator of the hypothalamic-pituitary-adrenal (HPA) axis, disruption of hippocampal integrity can result in unregulated activation of the stress response (13, 14). Clinical studies have correlated ELS with elevated resting and responsive cortisol levels in adulthood (11). High ACE exposure rates are associated with smaller hippocampal volumes in adults (15, 16), and the reduction in hippocampal volume appears to mediate the relationship between anxiety and ELS (17). In rodents, ELS disrupts synaptic potentiation within the CA3 layer of the hippocampus (18) and decreases neurogenesis in the dentate gyrus (19).

Recent studies in patients with Urologic Chronic Pelvic Pain Syndrome (UCPPS), which encompasses interstitial cystitis/painful bladder syndrome (IC/PBS) and chronic prostatitis/chronic pelvic pain syndrome (CP/CPPS), have uncovered symptomatic and central functional/connectivity features related to ELS exposure. Adult UCPPS patients exposed to ELS tend to report more widespread pain, more severe functional symptoms (painful urination), poorer self-perceived well-being, and were less likely to have symptom relief after a year follow up (7). UCPPS patients exposed to ELS also have altered regional cortical centrality patterns compared to both healthy controls and UCPPS patients without ELS exposure (20). These findings suggest that ELS results in distinct changes in brain functionality that may predispose patients to a worsened pain phenotype. Our laboratory has developed a clinically-relevant mouse model of ELS-induced chronic urogenital pain using neonatal maternal separation (NMS). Adult male and female NMS mice demonstrate urogenital hypersensitivity and dysfunction, widespread hypersensitivity, altered anxiety- and depression-like behaviors, increased adiposity, and reduced regulatory gene expression and neurogenesis in the hippocampus (21–27). Exposure to water avoidance stress (WAS) exacerbates many of the NMS-induced features, including gene expression within the hippocampus (21, 22, 28). Here we are using our NMS model in female mice to determine if ELS impacts hippocampal gray matter volume and its neurochemical responsiveness to WAS exposure using magnetic resonance imaging/spectroscopy (MRI/MRS) and voxel-based morphometry (VBM) analysis.

## Methods

### Animals

Experiments were performed on female C57Bl/6 mice (Charles River, Wilmington, MA) born and housed in the Research Support Facility at the University of Kansas Medical Center. Mice were housed at 22°C on a 12-h light cycle from 600 to 1800 h and received water and food *ad libitum*. All research performed conformed to the National Institute of Health Guide for the Care and Use of Laboratory Animals and was approved by the University of Kansas Medical Center Institutional Animal Care and Use Committee. To avoid exposing the mice to additional confounding stressors, no attempts were made to control or track the estrus cycle of the mice. In addition, our previous studies have shown no impact of cycle stage on other outcomes related to NMS exposure (22, 24, 29).

### Neonatal Maternal Separation

All mice used in this study were born in house from pregnant dams (Charles River, Wilmington, MA) delivered to the animal facility during the last week of gestation. The separation procedure was performed as previously described (23). Day of birth was designated as postnatal day (P) 0 and from P1 until P21 individual litters were removed daily and placed en masse into clean glass beakers containing a small amount of home cage bedding to maintain scent. Pups were held at 34°C and 50% humidity from 1100 to 1400 h. Fresh gloves were rubbed with home cage bedding before handling each litter to avoid rejection by the dam. Corresponding naïve mice were born, housed, and weaned during the same time frame to avoid potential complications arising from variations in prenatal shipping conditions, housing environment, and normal husbandry procedures. All mice were weaned on P22 and group housed with same-sex littermates. All litters also contained male pups, which were similarly handled, but not investigated in this study.

### Study Design and Timeline

Mice from the same cohort (naïve, *n* = 5; NMS, *n* = 9) were used for the reported results. The baseline and post-WAS MRI/MRS scans were performed 1 month apart at 8–9 months of age. Bladder sensitivity was evaluated in a subset of mice at 16 months of age. All mice remained on standard chow diet (8604; Harlan Teklad, Madison WI) and in group housing conditions with standard corncob bedding throughout the experiment.

### Body Composition Analysis

Prior to the baseline scan, mice were weighed and placed in an EchoMRI 2015 (EchoMRI LLC, Houston, TX) to quantify lean mass and fat mass.

### Water Avoidance Stress

Water avoidance stress (WAS) was performed for 1 h, within the first 6 h of the light cycle. Mice were placed individually on a round platform (5 cm diameter) centrally affixed to the bottom of a container (36 cm length × 31 cm width × 27 cm height) filled with room temperature tap water up to 1 cm below the top of the platform, as previously described (21, 22, 28).

### Magnetic Resonance Imaging (MRI) and Spectroscopy (MRS)

All Magnetic Resonance (MR) experiments were performed within the first 6 h of the light cycle on a 9.4 T MR system (Agilent Technologies, Santa Clara, CA) equipped with a 12 cm gradient coil (40 G/cm) (Magnex Scientific, Abingdon, UK). A custom-made quadrature surface radiofrequency (RF) coil consisting of two geometrically decoupled 18 mm loops and adjusted to transmit and receive at 400 MHz was used for acquisition of all MR data.

*In vivo* high-resolution T_2_-weighted MR images were acquired for brain volumetric analyses using a fast spin echo multi slice (FSEMS) sequence (TR = 4,000 ms, echo spacing = 0.012 ms, echo train length = 5, FOV = 2.56 × 2.56 cm^2^, matrix = 256 × 256, slice thickness = 0.2 mm, number of averages = 2, and total acquisition time = 6.25 m). Unbiased volumetric analysis of the whole brain was performed using Voxel Base Morphometry (VBM) implemented in SPM 8 and SPMMouse toolboxes within MATLAB (Mathworks Inc., Natick, MA) following published methodologies (30–32).

*In vivo* Magnetic Resonance Spectroscopy (MRS) was performed using a single-voxel SPECIAL sequence (33) (Volume of interest (VOI) = 2 × 1.2 × 1.8 mm^3^ positioned over the right hippocampus; TR = 4,000 ms; TE = 3 ms; Number of averages = 480; Total acquisition time = 32 m). The right hippocampus was chosen *a priori* due to clinical evidence illustrating unilateral functional defects in the right hippocampus in patients with anxious-depressive disorder related to changes in glucocorticoid receptor methylation patterns (34). Water suppression was performed with VAPOR (35) and linear and second order shims were automatically adjusted with FASTMAP (36) to achieve a water linewidth of no more than 18 Hz in a volume containing the VOI region. MR spectra was analyzed with LCModel software, using the unsuppressed water signal for each scan to calculate absolute metabolite concentrations (37, 38) and a metabolite basis set including alanine (Ala), ascorbate (Asc), aspartate (Asp), creatine (Cr), phosphocreatine (PCr), phosphocholine (PCho), glycerophosphocholine (GPC), gamma aminobutyric acid (GABA), glutamate (Glu), glutamine (Gln), glutathione (GSH), *myo*-inositol (mI), taurine (tau), lactate (Lac), N-acetylaspartate (NAA), *N*-acetylaspartylglutamate (NAAG), and phosphoethanolamine (PE) (full list found in Table 1). Total levels of choline (tCho) and NAA (tNAA) were also calculated and reported, as well as Glu+Gln (Glx).

**Table 1:** Hippocampal metabolite concentrations.

	**Naïve**		**NMS**	
	**Baseline**	**Post-WAS**	**Baseline**	**Post-WAS**
Alanine	0.97 ± 0.13	0.81 ± 0.38	1.03 ± 0.10	1.22 ± 0.16
**Ascorbate** **ω**	2.95 ± 0.29	3.42 ± 0.22	2.79 ± 1.0	3.22 ± 0.23
Aspartate	1.87 ± 0.21	1.68 ± 0.07	1.55 ± 0.12	1.66 ± 0.25
**tCholine §§ &**	1.89 ± 0.04	2.05 ± 0.06	1.68 ± 0.09	**1.58** ±0.09**
Creatine	5.35 ± 0.22	5.03 ± 0.27	5.25 ± 0.13	5.26 ± 0.322
Phosphocreatine	5.83 ± 0.21	6.34 ± 0.14	5.59 ± 0.20	5.40 ± 0.31
tCreatine	11.2 ± 0.13	11.4 ± 0.13	10.8 ± 0.20	10.7 ± 0.32
GABA	1.59 ± 0.17	1.76 ± 0.14	1.60 ± 0.11	1.63 ± 0.05
Glutamine	3.08 ± 0.11	3.31 ± 0.23	4.41 ± 0.77	4.78 ± 1.06
Glutamate	10.31 ± 0.18	10.32 ± 0.22	10.30 ± 0.22	10.31 ± 0.39
Glutathione	1.02 ± 0.12	1.21 ± 0.12	0.98 ± 0.05	1.02 ± 0.05
Lactate	2.93 ± 0.21	2.45 ± 0.13	2.99 ± 0.26	2.73 ± 0.24
*myo*-Inositol	7.06 ± 0.27	7.25 ± 0.14	6.30 ± 0.45	5.83 ± 0.50
**tNAA § &**	9.74 ± 0.38	10.0 ± 0.16	9.89 ± 0.10	**8.98** ±0.21**^#^
**PE** **ω*ω*** **&**	1.87 ± 0.21	**0.74** **±0.26**^**##**^	1.79 ± 0.16	**1.64** ±0.18*
Taurine	11.58 ± 0.26	11.40 ± 0.36	10.56 ± 0.32	10.73 ± 0.45
Glu/Gln (Glx)	13.40 ± 0.24	13.63 ± 0.33	14.71 ± 0.68	15.1 ± 0.89

Metabolite concentrations (μmol/g) were measured prior to (Baseline) and following a 1-h exposure to water avoidance stress (Post-WAS) in female naïve (n = 5) and NMS (n = 9) mice. Significant differences are indicated by bold text. § NMS effect (p < 0.05, 0.01), ω WAS effect (p < 0.05, 0.01), & NMS and WAS interaction effect (p < 0.05), two-way RM ANOVA; ^*^, ^**^p < 0.05, 0.01 vs. naïve, ^#^, ^##^p < 0.05, 0.01 vs. baseline, Bonferroni posttest. GABA, gamma aminobutyric acid; tNAA, N-acetylaspartate + N-acetylaspartylglutamate; PE, phosphoethanolamine; Glu, glutamate; Gln, glutamine.

### Urinary Bladder Distention

Mice were placed under inhaled isoflurane anesthesia (4% induction, 2% maintenance) and the bare ends of two Teflon-coated stainless steel electrode wires (0.003' diameter; Grass Technologies, West Warwick, RI) were inserted into the left and right abdominal musculature using a 26-guage needle. A 24-guage angiocatheter was inserted into the urethra and anchored to the tail using surgical tape. Anesthesia was lowered (~1%) until hindlimb reflexes, but not escape behaviors, were present. The bladder was distended with air from a compressed nitrogen tank equipped with a dual-stage low delivery pressure regulator (Matheson-Linweld, Kansas City, MO) controlled by a custom-made distention control device (The University of Iowa Medical Instruments, Iowa City, IA). A separate pressure monitor (World Precision Instruments, Sarasota, FL) was used to regulate the pressure within the bladder. Three 60 mmHg distentions were performed to establish stable responses and then each pressure (15, 30, 45, 60 mmHg) was applied in triplicate for 20 s with a 2-min rest period in between. Electromyographic (EMG) activity from the abdominal musculature was amplified, filtered, and recorded using Spike 2 software (Cambridge Electronic Design, Cambridge, UK) on a personal computer and analyzed off-line. The visceromotor response (VMR) was quantified by measuring the area under the curve of the distention period, divided by the duration of the distention, and expressed as a percent the baseline EMG activity immediately prior to the distention.

### Statistical Analysis

Calculations were made in Excel (Microsoft, Redmond, WA) and statistical analysis was performed using GraphPad Prism 9 (GraphPad, La Jolla, CA) or IBM SPSS Statistics 27 (IBM Corporation, Armonk, NY). Differences in volume between groups were determined by statistical parametric mapping in SPM 8 and SPM mouse. MRS and VMR results were analyzed via two-way ANOVA, with or without repeated measures, followed by Bonferroni posttest. Pearson correlation was used to correlate body weight and hippocampal spectral contents. All data are expressed as mean ± standard error of the mean (SEM). Statistical significance was set at *p* < 0.05.

## Results

### NMS Mice Have Smaller Left Hippocampal Volumes

**Figure 1:**
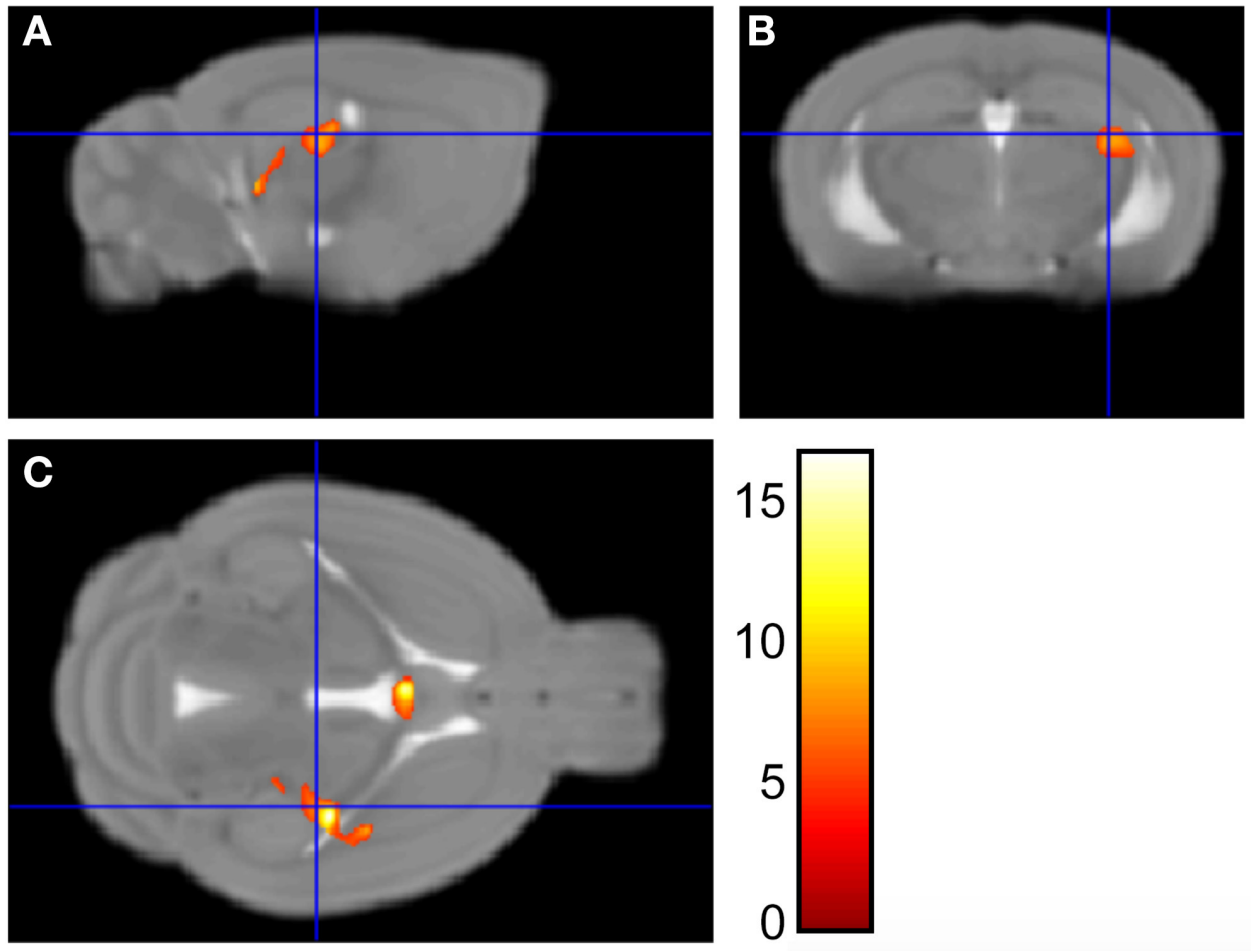
Voxel Based Morphometry analysis revealed regions that were significantly different in size between NMS (*n* = 9) and naïve (*n* = 5) female mice. Maximum intensity projections in the sagittal **(A)**, coronal **(B)**, and horizontal **(C)** plane are represented on the Mortimer Space Atlas, where the blue lines represent bregma corresponding to the left hippocampus.

We have previously published evidence of decreased hippocampal gene expression (21, 22, 24, 29) and neurogenesis (25) in NMS mice. Here, we are determining whether hippocampal volume is also impacted by NMS. Brain wide volume was analyzed using Voxel Based Morphometry (VBM) in female naïve and NMS mice prior to WAS exposure. The left hippocampal volume in the composite NMS brain was 0.038 mm^3^ smaller compared to naïve composite (*p* < 0.005) (Figure 1). We did not observe significant volume changes in the right hippocampus or in other identifiable structures in the brain (*p* > 0.05).

### NMS and WAS Significantly Impacted Hippocampal Neurochemical Concentrations

**Figure 2:**
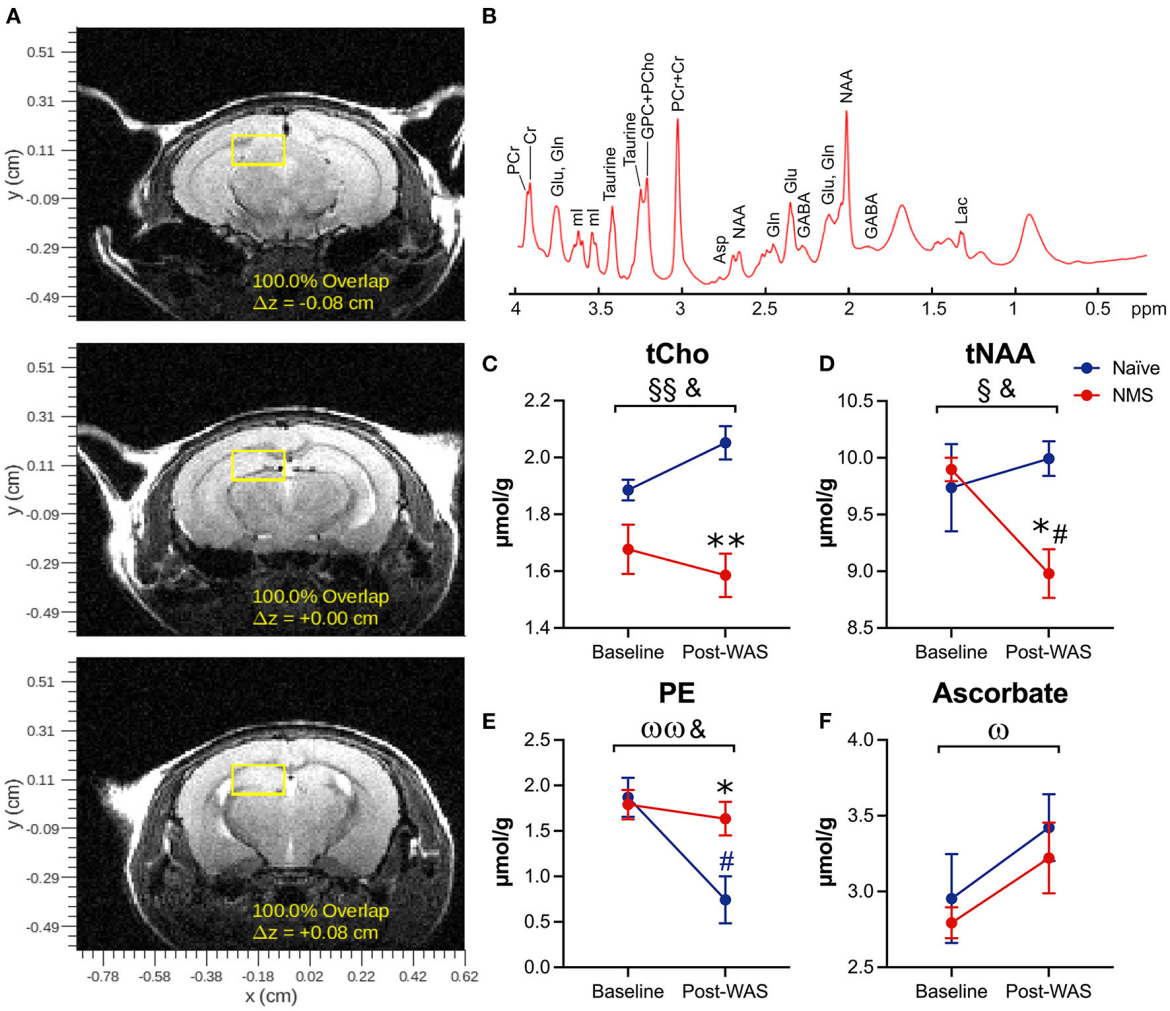
Magnetic Resonance Spectroscopy was performed on the right hippocampus prior to (baseline) and following water avoidance stress (Post-WAS) in naïve (*n* = 5) and NMS (*n* = 9) female mice. **(A)** Examples of the region of interest containing the right hippocampus for the collection of our MRS spectral data. From the resulting spectra **(B)** we measured absolute chemical concentrations. **(C)** A significant effect of NMS and an NMS/WAS interaction was observed on the level of total Choline (tCho). The post-WAS levels of tCho in NMS mice were significantly lower than in naïve mice. **(D)** A significant effect of NMS and an NMS/WAS interaction was observed on the combined level of N-acetylaspartate and *N*-acetylaspartylglutamate (tNAA). The level of tNAA in post-WAS NMS mice was significantly lower compared to both that in naïve mice and to their baseline measurements. **(E)** A significant effect of WAS and an NMS/WAS interaction was observed on phosphoethanolamine (PE). The level of PE in naïve mice, post-WAS, was significantly lower than in NMS mice and compared to their baseline measurements. **(F)** A significant effect of WAS was observed on the level of ascorbate. Brackets indicate a significant impact of NMS (§, §§*p* < 0.05, 0.01), WAS (ω, ωω*p* < 0.05, 0.01), or a NMS/WAS interaction effect (&*p* < 0.05) two-way RM ANOVA; *p < 0.05 vs. naïve, #*p* < 0.05 vs. baseline, Bonferroni posttest.

Single voxel MRS was used to measure the neurochemical content of only the right hippocampus prior to (baseline) and 24 h after a single exposure to WAS (Figure 2, Table 1). A significant reduction due to NMS was observed for total choline (tCho, *p* = 0.007) and tNAA levels (*p* = 0.044, Figure 2, Table 1). WAS exposure significantly decreased PE levels (*p* = 0.006) and significantly increased ascorbate levels (*p* = 0.026, Figure 2, Table 1). An interaction effect of NMS and WAS was observed for tCho (*p* = 0.029), tNAA (*p* = 0.035), and PE (*p* = 0.028, Figure 2, Table 1). Post-WAS, NMS mice had significantly decreased levels of tCho and tNAA compared to naïve mice and compared to baseline levels, only for tNAA (all *p* < 0.05, Figure 2, Table 1). PE levels were significantly lower post-WAS in naïve mice compared to NMS mice and baseline measurements (*p* < 0.05, Figure 2, Table 1). No significant differences were observed for the other measured chemicals (Table 1).

### Body Weight Was Correlated With Post-WAS Neurochemical Concentrations

**Figure 3:**
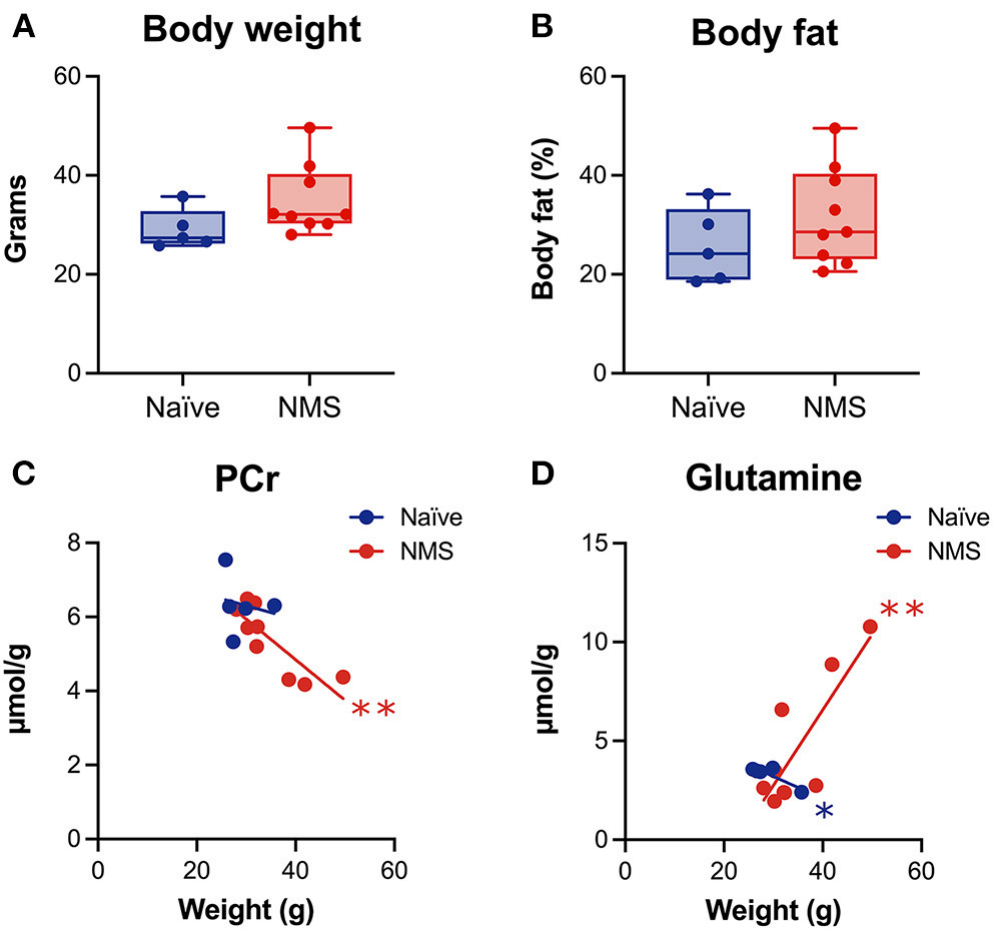
Body weight and percent body fat were measured prior to imaging and correlated with spectral contents after water avoidance stress exposure in naïve (*n* = 5) and NMS (*n* = 9) female mice. A non-significant increase in body weight **(A)** and percent body fat **(B)** was observed in female NMS mice compared to naïve mice. **(C)** A significant negative correlation was observed between hippocampal phosphocreatine (PCr) levels and body weight in NMS mice. **(D)** Significant negative and positive correlations were observed in naïve and NMS mice, respectively, between hippocampal glutamine levels and body weight. *, **p < 0.05, 0.01, Pearson correlation.

We have previously published evidence of increased body weight and fat mass in adult NMS mice (26, 27), however, these changes have not been investigated in conjunction with alterations in the hippocampus. Prior to the baseline scans, the body weight and body fat percentage (Figure 3) of NMS mice were slightly higher than naïve mice. When baseline body weight was correlated to post-WAS neurochemical concentrations in the hippocampus, there was a significant negative correlation for PCr in NMS mice (*p* = 0.0051, Figure 3) and a significant negative correlation for Glutamine in naïve mice (*p* = 0.0467), while NMS mice showed a positive correlation for Glutamine (*p* = 0.007, Figure 3). A significant negative correlation was also observed for body fat and PCr in NMS mice (*p* = 0.001, data not shown). No significant correlations were observed for the other measured chemicals.

### Bladder Sensitivity Was Significantly Increased in NMS Mice

**Figure 4:**
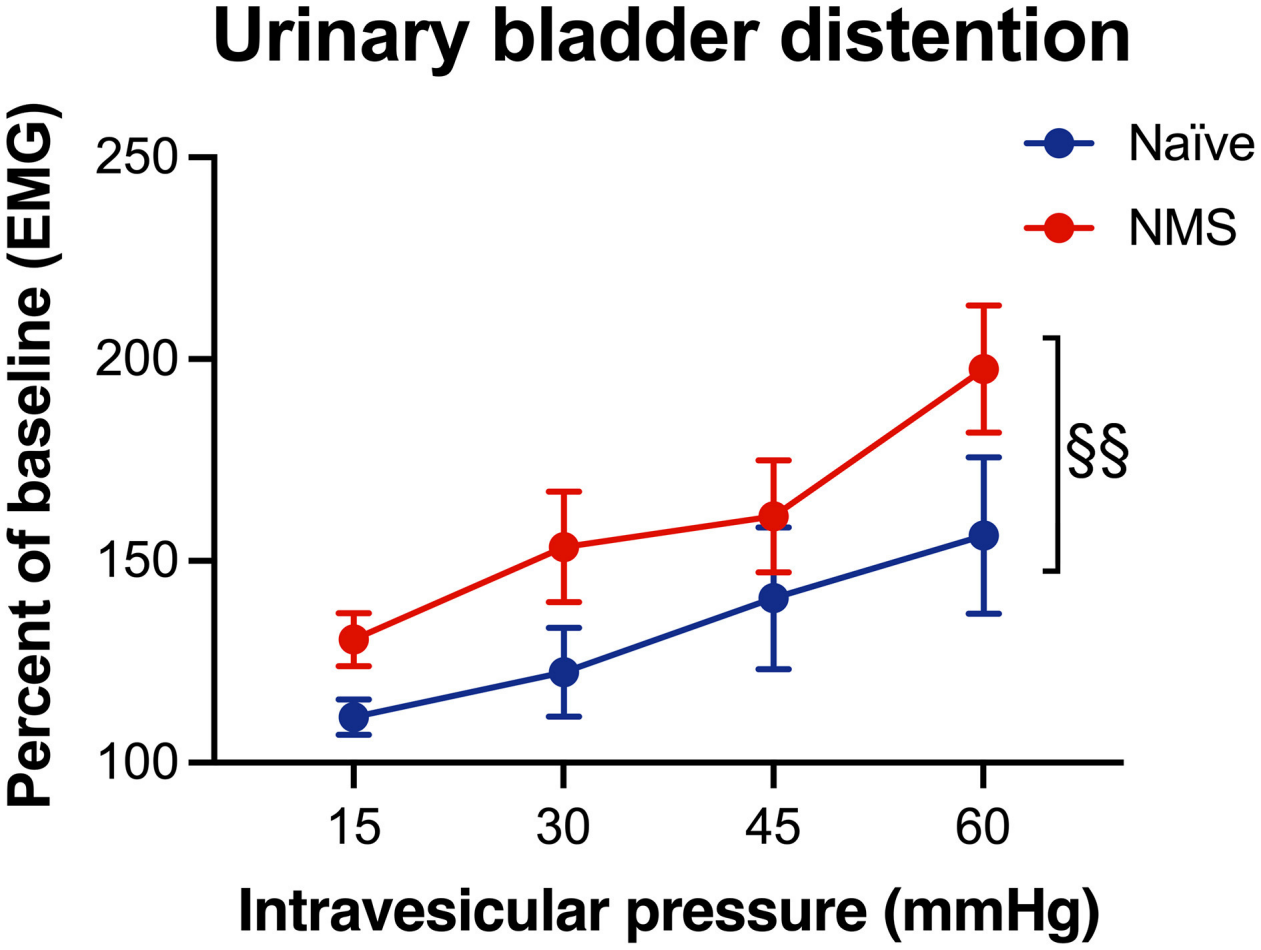
Visceromotor response (VMR) during urinary bladder distention (UBD) was measured seven months after water avoidance stress exposure and MR imaging in naïve and NMS female mice. An increase in VMR during UBD was observed in NMS mice (*p* = 0.0094) compared to naïve mice. Two-way ANOVA with Bonferroni posttest. Naïve, *n* = 5, NMS, *n* = 7.

To verify that NMS increased urogenital hypersensitivity, a subset of mice were assessed for their visceromotor response (VMR) during urinary bladder distention (UBD). As previously reported (22, 29), we observed a significant increase in VMR during UBD in the female NMS mice compared to naïve mice (*p* = 0.0094, Figure 4).

## Discussion

This study examined the effects of ELS, in the form of NMS, on hippocampal volume as well as hippocampal neurochemical concentrations before and after an acute stressor in adult female mice. We also determined the potential impact of body weight on post-WAS hippocampal neurochemical content, as we have previously observed that NMS increases body weight and fat content. We demonstrated that NMS mice had reduced volume in the left hippocampus. NMS significantly reduced the levels of several neurochemicals in the hippocampus, particularly after WAS exposure, which supports our previous work showing decreased hippocampal gene expression (21, 22, 24, 25, 29) and neurogenesis (25) in NMS mice.

Voxel based morphometry (VBM), while a very popular tool in human research, is less frequently used in rodent research. With the development of rodent compatible software and the use of higher-powered MRI, VBM is capable of whole brain gray and white matter volume comparisons in rodents (31, 32). Importantly, VBM avoids the human error of atlas-based manual measurements, which are prone to subjective user variability. Furthermore, unlike region of interest-based MRI volume analysis, VBM is not limited to studying a single region. As such, VBM allows for a brain-wide analysis of gray or white matter volume that can detect subtle differences in structural volume (30, 39, 40). Implemented here, VBM allowed for comparisons of whole brain gray matter volume to identify any significant changes between NMS and naive mice. We observed significantly reduced volume in the left hippocampus of NMS mice, compared to naïve mice. The hippocampal finding is clinically important as patient-reported ELS is associated with decreased hippocampal gray matter volume (15, 41–45), which is also a common feature of patients with anxious-depressive disorders (34, 45–47). Interestingly, clinical findings reported gray matter losses specifically in the left hippocampus, whereas changes in functional connectivity appear to affect the right hippocampus (34). The developing hippocampus is particularly sensitive to excess glucocorticoids and overexposure can permanently affect hippocampal structure, function, and gene expression (12). Rodent models of ELS have shown delayed maturation of the dentate gyrus (DG), resulting in fewer neural stem cells in the adult DG (48). We have previously shown reduced hippocampal neurogenesis in male NMS mice, compared to naïve mice (25), and our results here suggest that similar reductions may be occurring in the female mice, as well.

We have previously shown that exposure to WAS significantly increased urogenital sensitivity and impacted bladder output in both male and female NMS mice, along with concordant gene expression changes in the hypothalamus, amygdala, and hippocampus (21, 22). Although in the previous studies we observed hippocampal gene expression changes up to 8 days post-WAS, here we used MRS, a non-invasive imaging technique, to determine neurochemical abnormalities in the hippocampus 1 day after WAS exposure to examine more immediate changes due to acute stress exposure. Although relatively few MRS studies have looked at the impact of ELS, studies of chronic and acute adult stress exposure have shown neurochemical changes within the hippocampus and other related structures. NAA is a marker of neuronal integrity and viability, and is an indicator of neuronal mitochondria function (49). Bi-lateral decreased hippocampal NAA was observed in adult males with an early history of socioeconomic deprivation (50). Lower hippocampal NAA concentrations have also been repeatedly demonstrated in patients diagnosed with PTSD (51) and major depressive disorder (52), with or without a concordant loss of gray matter volume. Here we observed a significant decrease in tNAA due to NMS, as well as an NMS/WAS interaction. NMS mice had a significantly lower level of tNAA after WAS exposure, compared to their baseline measurements and to WAS-exposed naïve mice. In addition, PE is an ethanolamine derivative involved in phospholipid formation and has been implicated in mitochondrial dysfunction contributing to depressive disorders (53). Previous studies have reported increased levels of PE in the brains of mice that were exposed to chronic unpredictable stress to model depression (54). We observed a significant impact of WAS on PE expression in naïve mice, although it remained unchanged in NMS. Together these observations suggest that there may be mitochondrial dysfunction in the hippocampus of NMS mice, which could lead to reduced resilience and inappropriate response to an acute stressor.

The level of tCho, which consists of glycerophosphocholine (GPC) and phosphocholine (PCho), was also significantly decreased in the right hippocampus of NMS mice. PCho is a phosphomonoester and its concentration has been positively associated with dendritic sprouting, such as that occurring during development, in response to cortical lesion, and in early Alzheimer's disease progression (55). It is also a precursor for phosphatidylcholine, which is highly enriched in the hippocampus and its homeostatic regulation is vital for maintaining membrane trafficking and axonal integrity (56). The diminished level of tCho in the hippocampus of NMS mice was further exacerbated following WAS exposure, again suggesting that NMS may have diminished the neuronal integrity of the hippocampus.

Ascorbate is an endogenous antioxidant that is highly expressed in the brain, particularly in the amygdala, hypothalamus, and hippocampus (57), which are the main initiators and regulators of the stress response system (3). Ascorbate is neuroprotective and prevents injury from ischemia and excitotoxicity (57). We interpret the increase in ascorbate levels to be a neuroprotective mechanism in response to WAS exposure. The equivalent response of both groups suggests that general oxidative stress in not contributing to the NMS-related outcomes.

Adults who report high adverse childhood events (ACE) scores are more likely to present with obesity and/or metabolic syndrome (58). The brain is susceptible to obesity-related pathology, including endothelial damage and more pronounced age-related volume reductions (59, 60). Patients with bipolar disorder have a 60% higher obesity rate, which is also related to decreased hippocampal volume and lower neurochemical levels, including that of PCr and PCho (61). PCr is a high-energy phosphate that serves as an intracellular energy buffer supporting mitochondrial respiration, and also exhibits neuroprotective effects through reduction of reactive oxygen species (62). Several clinical MRS studies have found altered brain PCr concentrations in schizophrenia, depression, panic disorder, and substance abuse patients (63–65). Although, we only observed a trend toward increased body weight and fat in the NMS mice, there was a significant negative correlation between body weight and body fat with PCr, suggesting that hippocampal expression of this important metabolic compound may be linked to adiposity. A positive relationship between body mass index (BMI) and hippocampal Glx (glutamate/glutamine) was also reported for patients with bipolar disorder (66), however the opposite was observed in rats fed a high fat diet (67). While we did not observe a significant relationship between body weight and Glx in our study, we did observe a negative correlation between body weight and glutamine for naïve mice and a positive correlation for NMS mice. Glutamate is the most abundant excitatory neurotransmitter in the brain and is taken up by astrocytes and converted to glutamine following synaptic release (66). Leptin receptors on astrocytes in the hippocampus have been shown to regulate glutamate homeostasis and neurotransmission (68). High fat diet has also been shown to impact both the morphology of and increase the expression of glutamate transporters on astrocytes (69). Together, these correlative outcomes suggest that whole body metabolism may be influencing neuronal-glial communications and local synaptic activity within the hippocampus, particularly in response to acute stress exposure.

We acknowledge a number of limitations within our study that may affect the impact of our results. First, we only investigated female mice for the current study. Based on our previous observations of decreased hippocampal gene expression and neurogenesis following NMS (21, 25), we predict that similar changes would be observed in male mice and studies are currently underway to determine any sex differences in these outcomes. Second, we were only able to perform single voxel MRS and a priori chose the right hippocampus due to clinical evidence of diminished unilateral hippocampal connectivity following ELS (34) and prior to completed VBM analysis of the MRI results. We do not report these results as evidence of unilateral neurochemical changes in the hippocampus and our ongoing studies will incorporate bi-lateral MRS analysis of the hippocampus to determine if NMS and WAS effects are observed in both hippocampi. Finally, our groups were not of even number and the naïve group may have been underpowered at *n* = 5.

Our results reported here support our previous findings of impaired hippocampal integrity following NMS exposure in mice. The left hippocampus of NMS mice was significantly smaller than naïve mice and single voxel MRS of only the right hippocampus revealed that it was less metabolically sensitive to acute stress exposure. In addition, correlations between body weight and neurochemical content within the hippocampus suggest that whole body metabolic state may influence neuronal-glial communication and synaptic function within the hippocampus, or perhaps vice versa. Understanding the effects, and directionality, of ELS on metabolic and structural changes in the brain and accompanying urogenital and metabolic outcomes, will provide important mechanistic insight toward developing preventative measures and/or treatment options for patients with chronic pain, mood, and metabolic disorders and a history of ELS.

## Data Availability Statement

The raw data supporting the conclusions of this article will be made available by the authors, without undue reservation.

## Ethics Statement

The animal study was reviewed and approved by the University of Kansas Medical Center Institutional Animal Care and Use Committee.

## Author Contributions

I-YC, JH, and JC designed the research study. AB, XY, C-YL, PL, PK, and OE performed the experiments. AB, XY, C-YL, PL, PK, I-YC, JH, and JC analyzed the data. AB and JC wrote the manuscript. All authors contributed to the article and approved the submitted version.

## Funding

This work was supported by NIH grants R01 DK099611 (JC), R01 DK103872 (JC), the KUMC Research Institute, Lied Basic Science Grant Program (JC), Center of Biomedical Research Excellence (COBRE) grant P20 GM104936 (JC), T32 HD057850 (AB and OE), start-up funds and core support from the Kansas Institutional Development Award (IDeA) P20 GM103418, and core support from the Kansas IDDRC P30 HD00228.

## Conflict of Interest

The authors declare that the research was conducted in the absence of any commercial or financial relationships that could be construed as a potential conflict of interest.

## Publisher's Note

All claims expressed in this article are solely those of the authors and do not necessarily represent those of their affiliated organizations, or those of the publisher, the editors and the reviewers. Any product that may be evaluated in this article, or claim that may be made by its manufacturer, is not guaranteed or endorsed by the publisher.
